# Supplementary figures and images for: Sixteenth-century tomatoes in Europe: who saw them, what they looked like, and where they came from
Source: PeerJ. 2022 Jan 17;10:e12790. doi: 10.7717/peerj.12790 (PMC8772448; doi:10.7717/peerj.12790)

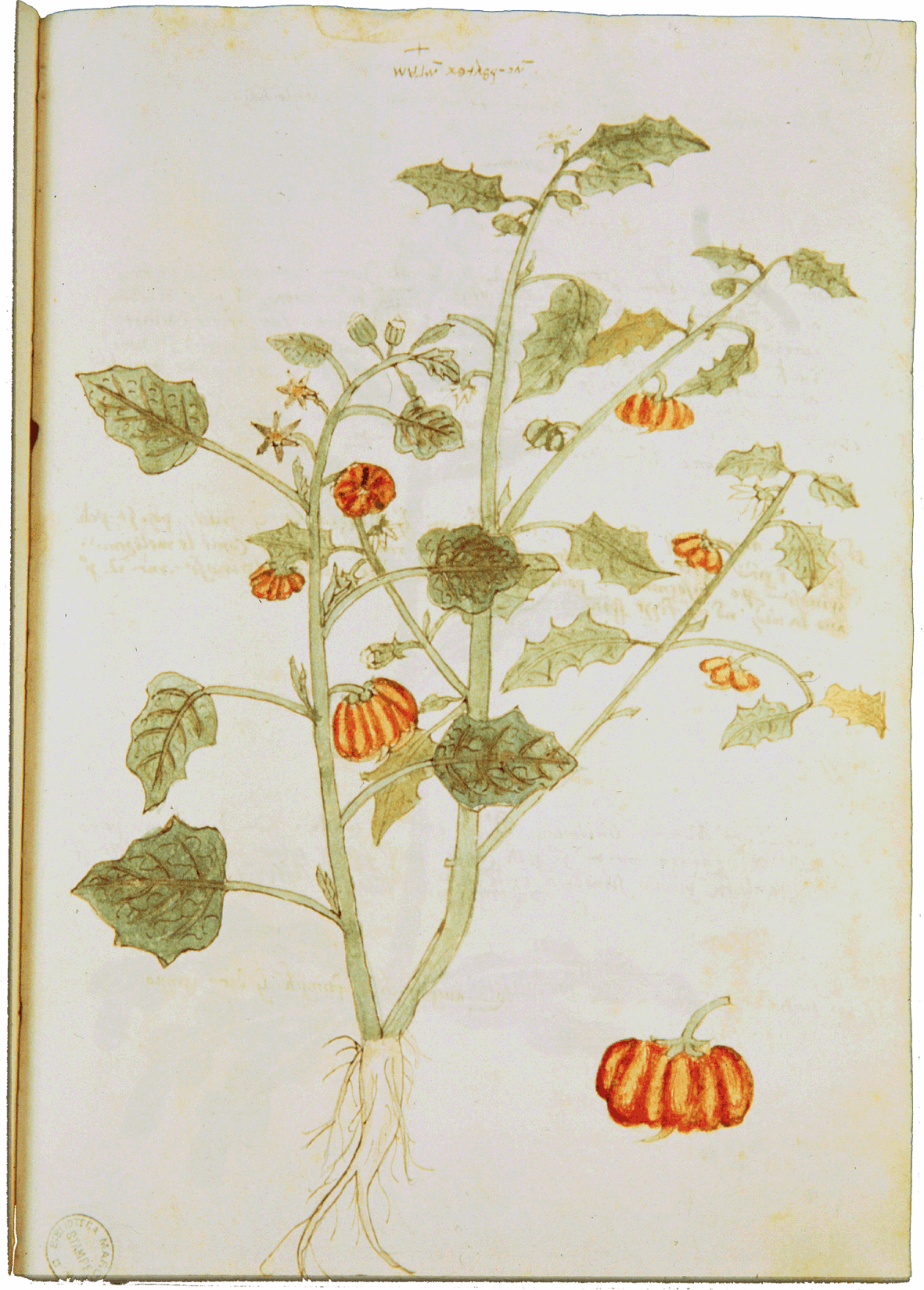

Supplement: Supplemental Information 2 — Artist unknown, manuscript: I cinque libri di piante. Codice Marciano, 1551-1575. Image credit: Biblioteca Marciana, Venice. [file peerj-10-12790-s002.jpg]

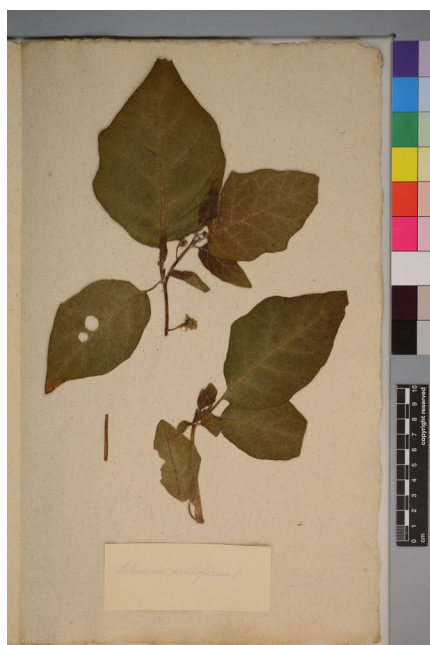

Supplement: Supplemental Information 3 — Image credit: University of Basel. [file peerj-10-12790-s003.pdf]
